# Identification of acidic stress-responsive genes and acid tolerance engineering in *Synechococcus elongatus* PCC 7942

**DOI:** 10.1007/s00253-023-12984-5

**Published:** 2024-01-10

**Authors:** Jie Zhang, Tao Sun, Weiwen Zhang, Lei Chen

**Affiliations:** 1 Laboratory of Synthetic Microbiology, School of Chemical Engineering & Technology, Tianjin University, Tianjin, 300072 People’s Republic of China; 2 Frontiers Science Center for Synthetic Biology, Tianjin University, Tianjin, 300072 People’s Republic of China; 3 Key Laboratory of Systems Bioengineering (MOE), Tianjin University, Tianjin, 300072 People’s Republic of China; 4 Center for Biosafety Research and Strategy, Tianjin University, Tianjin, 300072 People’s Republic of China

**Keywords:** Acid tolerance, Transcriptomics, Cyanobacteria, Chassis, Engineering target

## Abstract

**Abstract:**

Cyanobacteria are excellent autotrophic photosynthetic chassis employed in synthetic biology, and previous studies have suggested that they have alkaline tolerance but low acid tolerance, significantly limiting their productivity as photosynthetic chassis and necessitating investigations into the acid stress resistance mechanism. In this study, differentially expressed genes were obtained by RNA sequencing-based comparative transcriptomic analysis under long-term acidic stress conditions and acidic shock treatment, in the model cyanobacterium *Synechococcus elongatus* PCC 7942. A pathway enrichment analysis revealed the upregulated and downregulated pathways during long-term acidic and shock stress treatment. The subsequent single gene knockout and phenotype analysis showed that under acidic stress conditions, the strains with *chlL*, *chlN*, *pex*, *synpcc7942_2038*, *synpcc7942_1890*, or *synpcc7942_2547* knocked out grew worse than the wild type, suggesting their involvement in acid tolerance. This finding was further confirmed by introducing the corresponding genes back into the knockout mutant individually. Moreover, individual overexpression of the *chlL* and *chlN* genes in the wild type successfully improved the tolerance of *S. elongatus* PCC 7942 to acidic stress. This work successfully identified six genes involved in acidic stress responses, and overexpressing *chIL* or *chIN* individually successfully improved acid tolerance in *S. elongatus* PCC 7942, providing valuable information to better understand the acid resistance mechanism in *S. elongatus* PCC 7942 and novel insights into the robustness and tolerance engineering of cyanobacterial chassis.

**Key points:**

*• DEGs were identified by RNA-seq based transcriptomics analysis in response to acidic stress in S. elongatus PCC 7942.*

*• Six genes were identified to be involved in acid tolerance in S. elongatus PCC 7942.*

*• Overexpression of chIL or chIN individually successfully improved the acid tolerance of S. elongatus PCC 7942.*

**Supplementary information:**

The online version contains supplementary material available at 10.1007/s00253-023-12984-5.

## Introduction

Photoautotrophic cyanobacteria, distributed in almost every conceivable habitat, play an essential role in global CO_2_ and N_2_ fixation, and ecosystem stability (Parmar et al. 2011). Moreover, in recent decades, synthetic biology research has also led to the development of photosynthetic cyanobacteria as “autotrophic cell factories” for the biosynthesis of various biofuels and chemicals directly from CO_2_, including 2, 3-butanediol, lactate, isobutanol, and ethanol (Kelly et al. 2018). However, ubiquitous acidic environments, such as acidified lakes, streams, and acid rain, significantly influence the primary productivity of cyanobacteria (Uchiyama et al. 2014). For example, in *Synechocystis* sp*.* PCC 6803, different pH conditions have been shown to significantly affect cell growth performance (Ohta et al. 2005). This cyanobacterium grows advantageously under alkaline conditions, and the growth rate gradually increases when the pH ranges from 4.4 to 7.7; it cannot survive at a pH below 4.4 (Huang et al. 2002). Cyanobacteria have been reported to produce various biofuels with high yields; for example, *Synechococcus elongatus* PCC 7942 could synthesize biofuel ethanol with a high yield of 3856 mg/L (Velmurugan et al. 2020). However, further increasing their productivity remains a major challenge, and the improvement of cellular robustness, such as resistance to acidic stress conditions, will greatly benefit the environmentally friendly applications of these important cyanobacterial chassis (Kumar et al. 2022). Cyanobacteria responses to acidic stress involve many complex reaction mechanisms. A previous study suggested that All5304, part of an efflux pump, was necessary for acid tolerance by comparing the exoproteome in *Anabaena* sp. PCC 7120 (Shvarev and Maldener 2020)*.* In another study, the acid resistance mechanism at the genetic level in *Synechocystis* sp. PCC 6803 was explored, and the result indicated that Sll0751 and Sll1041 of ABC transporter subunits were involved in acidic stress responses (Tahara et al. 2015). Meanwhile, whole-genome sequencing of adaptively evolved cells revealed 11 mutations involved in acid tolerance in *Synechocystis* sp*.* PCC 6803; however, the mechanism was unclear (Uchiyama et al. 2015). Additionally, the cellular functionality of cyanobacteria is influenced by the pH within a specific range, and acidic stress affects diverse biological processes, encompassing cell wall biosynthesis (Rezayian et al. 2019). Therefore, deciphering the tolerance mechanism to acidic stress in cyanobacteria is critical.

**Fig. 1:**
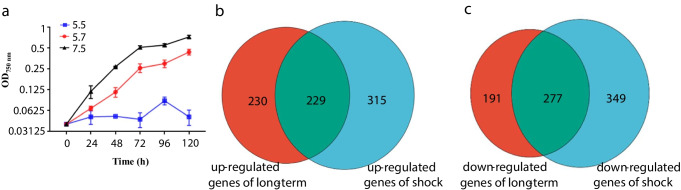
Growth pattern of *S. elongatus* PCC 7942 and DEGs (fold change ≥ 2 and *p* ≤ 0.05) among long-term acidic stress, acid shock stress, and control conditions. **a** Growth pattern in BG11 at pH 7.5, 5.7, and 5.5;** b** up-regulated genes after long-term stress vs. control and shock treatment vs. control;** c** down-regulated genes after long-term stress vs. control and shock treatment vs. control. The error bars represent the calculated standard deviation of the measurements of three biological replicates

*S. elongatus* PCC 7942 is a model prokaryotic unicellular cyanobacterium in which many genetic editing tools have been developed (Andersson et al. 2000). This model species is commonly used for studying photosynthesis and circadian rhythms, and it has also been developed for the bioproduction of various biofuels, chemicals, and pharmaceuticals (Yin et al. 2019). However, *S. elongatus* PCC 7942 is highly sensitive to acidic stress. Cell growth is nearly halted at a pH of 5.5, severely constraining its economic viability as an autotrophic chassis. Alternatively, at a pH of 5.7, cell growth is sluggish, making it suitable for studying the impact of acidity on cellular behavior (Fig. 1a). To date, few studies have been conducted on the mechanisms of resistance against acidic stress and engineering of acidic tolerance in *S. elongatus* PCC 7942. Therefore, it is necessary to decipher the resistance mechanism against acidic stress in *S. elongatus* PCC 7942. This investigation will facilitate rational engineering to improve its acidic tolerance and lay a foundation for a better understanding of the resistance mechanism against acidic stress in cyanobacteria.

RNA sequencing (RNA-seq) is currently used to reveal transcriptome differences, including mRNA, rRNA, and tRNA, in different treatments or stages of the same cells (Haque et al. 2017). In this study, to reveal the resistance mechanism against acidic stress in *S. elongatus* PCC 7942, RNA-seq was employed to explore differentially expressed genes (DEGs) under acidic stress, revealing six acidic stress-responsive genes. This study demonstrated the feasibility of employing comparative transcriptomics in stress resistance mechanism investigations in cyanobacteria and lays a foundation for cyanobacterial robustness and engineering applications.

## Materials and methods

### Bacterial growth conditions and acid stress treatment

*S. elongatus* PCC 7942 and the strains constructed in this study were cultivated in BG11 medium under a light intensity of approximately 50 μmol photos m^−2^ s^−1^ in an HNY-211B Illuminating incubator shaker at 180 rpm and 37 °C (Honour, Tianjin, China). Cell density was measured at OD_750 nm_ using an ELx808 Absorbance Microplate Reader (BioTek, Winooski, VT, USA). Long-term acidic stress treatment and acidic shock were carried out by adding 4-morpholineethanesulfonic acid hydrate (MES) (Merck, M5287, Darmstadt, Germany) to the medium. All samples were collected by centrifugation (8000 × *g*, 10 min, at 4 °C) and subsequently subjected to RNA-seq analysis.

### Transcriptomic analysis

Cells were cultured in BG11 medium with a beginning pH of 5.7 supplemented with MES for 60 h, with BG11 medium at pH 7.5 as a control to investigate long-term acidic stress conditions. For acidic shock treatment, cells were cultured in normal BG11 medium at pH 7.5 for 60 h, followed by the addition of HCl to adjust the pH to 5.7 and were then further cultured for 1 h before harvesting. Treated and control cells were collected at the cultivation time points of 60 h for long-term acidic stress treatment and 1 h after acidic shock treatment. Samples were collected and sent to Novogene (Beijing, China) for transcriptome sequencing and data analysis. Three biological replicates were produced per sample. A fold change ≥ 2 and a *p* value ≤ 0.05 were set as the thresholds for DEG identification.

### qRT‒PCR analysis

qRT‒PCR was used to determine the relative expression level of genes under normal and acidic stress conditions. The primers for qRT‒PCR analysis were designed by Primer Express 2.0, and they are listed in Table S1. The qPCR was achieved using ChamQ Universal SYBR qPCR Master Mix (Vazyme Biotech, Q711, China), and three technical replicates were performed per sample. Step One Plus analytical software was used for the data analysis (Applied Biosystems, Foster City, CA, USA), and the 2^−ΔΔCT^ method was employed for calculations (Livak et al. 2001). 16S rRNA was selected as an internal reference. The data are presented as the ratio of the number of gene transcripts in cells under long-term acidic stress and acidic shock stress compared to that under control conditions.

### Strain construction

The strains used in this study are listed in Table 1. For gene knockout, upstream and downstream homologous arms were amplified from the genome of *S. elongatus* PCC 7942 and ligated with a chloramphenicol-resistance cassette (amplified from the plasmid pACYC184) by fusion PCR. The gene ORF and a spectinomycin-resistant cassette were created using the previously constructed plasmid pSI-*cpc560*-*lacZ* (Li et al. 2018) to achieve gene complementation and overexpression. These constructs were then inserted between the upstream and downstream homology arms of neutral site (NS) I. The primers used in this study are listed in Table S1, and they were synthesized by Azenta (Suzhou, China). The target fragments were amplified by Phanta Super-Fidelity DNA Polymerase (Vazyme Biotech, P505, China) and purified using a Cycle Pure Kit (Omega, D6492, USA).

**Table 1:** Strains used in this study

Name	Description	References
WT	*Synechococcus elongatus* PCC 7942	Laboratory storage
∆*chlL*	WT-∆*chlL*::*cm*^r^	This study
∆*chlN*	WT-∆*chlN*::*cm*^r^	This study
∆*pex*	WT-∆*pex*::*cm*^r^	This study
∆*synpcc7942_2038*	WT-∆*synpcc7942_2038*::*cm*^r^	This study
∆*synpcc7942_1890*	WT-∆*synpcc7942_1890*::*cm*^r^	This study
∆*synpcc7942_2547*	WT-∆*synpcc7942_2547*::*cm*^r^	This study
∆*chlL-*com	∆*chlL*-NS1::* P*_*cpc560*_*_ chlL _T*_*rbcl*_* spe*^*r*^	This study
∆*chlN-*com	∆*chlN*- NS1::* P*_*cpc560*_*_ chlN _T*_*rbcl*_* spe*^*r*^	This study
∆*pex*-com	∆*pex*- NS1::* P*_*cpc560*_*_ pex _T*_*rbcl*_* spe*^*r*^	This study
*∆synpcc7942_2038-*com	∆*synpcc7942_2038*-NS1::* P*_*cpc560*_*_ synpcc7942_2038 _T*_*rbcl*_* spe*^*r*^	This study
*∆synpcc7942_1890-*com	∆*synpcc7942_1890*-NS1::* P*_*cpc560*_*_ synpcc7942_1890 _T*_*rbcl*_* spe*^*r*^	This study
*∆synpcc7942_2547-*com	∆*synpcc7942_2547*-NS1::* P*_*cpc560*_*_ synpcc7942_2547 _T*_*rbcl*_* spe*^*r*^	This study
OE-*chlL*	NS1::* P*_*cpc560*_*_ chlL _T*_*rbcl*_* spe*^*r*^	This study
OE-*chlN*	NS1::* P*_*cpc560*_*_ chlN _T*_*rbcl*_* spe*^*r*^	This study
OE-*pex*	NS1::* P*_*cpc560*_*_ pex _T*_*rbcl*_* spe*^*r*^	This study
OE-*synpcc7942_2038*	NS1::* P*_*cpc560*_*_ synpcc7942_2038 _T*_*rbcl*_* spe*^*r*^	This study
OE-*synpcc7942_1890*	NS1::* P*_*cpc560*_*_ synpcc7942_1890 _T*_*rbcl*_* spe*^*r*^	This study
OE-*synpcc7942_2547*	NS1::* P*_*cpc560*_*_ synpcc7942_2547 _T*_*rbcl*_* spe*^*r*^	This study
OE-con	NS1::* P*_*cpc560*_* _T*_*rbcl*_* spe*^*r*^	This study

The natural transformation of *S. elongatus* PCC 7942 was performed following a previously reported method with some modifications (Onai et al. 2004; Pope et al. 2020). In brief, a 5 mL culture of exponentially growing *S. elongatus* PCC 7942 (OD_750 nm_ ≈ 0.8) was centrifuged (8000 × *g*, 5 min, at 4 °C), and the pellet was washed with 10 mM NaCl (4000 × *g*, 10 min, at 4 °C). The supernatant was removed, and the sample was resuspended in 200 μL of BG11 medium containing the target fragments (1000–1500 ng). The cell-DNA mixtures were spread on BG11 agar plates with sterile filters (0.45 µm pore size) and incubated in the dark at 30 °C for 10 h. Subsequently, the filter was transferred to a fresh BG11 agar plate supplemented with appropriate antibiotic(s) (10 μg/mL chloramphenicol, 25 μg/mL spectinomycin). Clones were observed after 5–7 days of incubation under an intensity of approximately 200 μmol photons m^−2^ s^−1^.

### Acid tolerance analysis

To monitor the growth profile under acidic stress, WT and constructed strains were collected by centrifugation (8000 × *g*, 10 min, at 4 °C), and the initial concentration of cells at OD_750 nm_ of 0.1 was prepared and inoculated into 20 mL of BG11 liquid medium for 12 h in a 100-mL flask. For growth patterns, 4 mL of fresh cells at OD_750 nm_ of 0.2 was collected by centrifugation (8000 × *g*, 10 min, at 4 °C) and then inoculated into 20 mL of BG11 liquid medium (normal or acid stress) in a 100-mL flask with OD_750 nm_ of 0.04. All culture samples were taken and measured at OD_750 nm_ every 12 h. Three biological parallels were used for each sample.

## Results

### Acidic stress responses revealed by RNA-seq based transcriptomics analysis in *S. elongatus* PCC 7942

The acid sensitivity of *S. elongatus* PCC 7942 was first evaluated to explore the DEGs and investigate the resistance mechanism against acidic stress in *S. elongatus* PCC 7942. The growth of *S. elongatus* PCC 7942 under an initial cultivation pH of 5.5, 5.7, and 7.5 was observed. The results showed that *S. elongatus* PCC 7942 barely survived at an initial cultivation pH of 5.5, and a significant growth decrease was observed at pH 5.7 compared to that at pH 7.5 (Fig. 1a). Thus, cells exposed to a pH of 5.7 were selected for the subsequent comparative transcriptomics analyses (long-term acidic stress condition, cells collected at time point 60 h) and the 1 h acidic shock treatment at a cultivation time point of 60 h.

For the RNA-seq-based transcriptomics analysis, approximately 15 million clean reads were obtained with Q30 > 94% and an error rate ≈ 0.02% per sample. The correlation of gene expression levels was evaluated, and the results showed good biological reproducibility, with three distinct clusters formed in the principal component analysis (PCA) (Patro et al. 2014) (Fig. S1).

Using the criteria of fold change ≥ 2 and *p* ≤ 0.05, a total of 927 DEGs were identified, including 459 up-regulated genes and 468 down-regulated genes (versus the control) under long-term acidic stress conditions, and a total of 1170 DEGs, including 544 up-regulated genes and 626 down-regulated genes (versus the control), were identified under acidic shock treatment (Tables S2 and S3). Among the identified DEGs above, 229 up-regulated genes and 277 down-regulated genes were shared among the long-term acidic stress and shock stress-responsive genes (Fig. 1b, 1c). The shared DEGs under both acidic stress conditions are good candidate engineering targets for acid tolerance modification, given their involvement in acid tolerance according to the two tested acidic stress conditions in this study. However, individual DEGs under the two tested stress conditions that did not overlap were also important, suggesting that the cells adapted different strategies to combat long-term acidic and acidic shock stress.

Seven genes were analyzed by qRT‒PCR to evaluate the reliability of the gene expression levels obtained from the RNA-seq analysis (Table S4). As shown in Fig. S2, a good correlation was observed between the expression levels obtained from qRT‒PCR and RNA-seq, with *R*^2^ = 0.757 (long-term acidic stress) and 0.805 (acidic shock stress), suggesting the good reliability of the RNA-seq data.

**Fig. 2:**
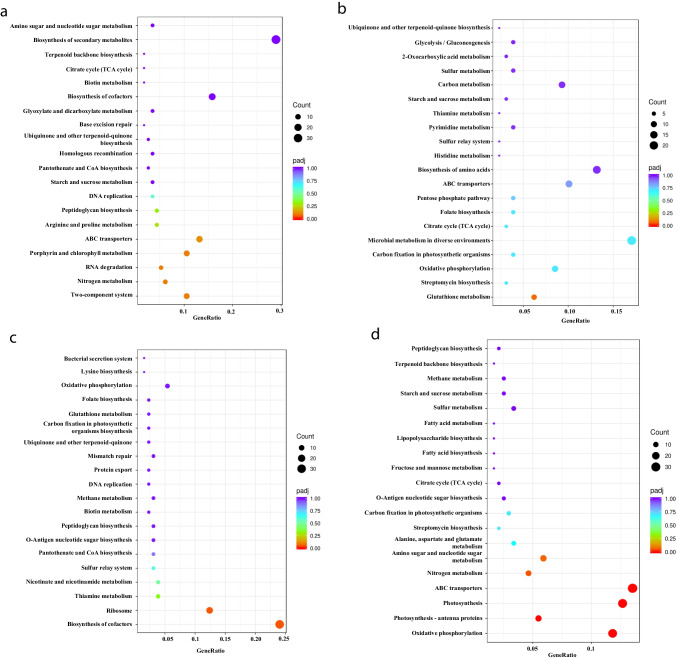
Scatter plot of KEGG pathway enrichment analysis. **a** Enriched pathways among the up-regulated DEGs associated with long-term acidic stress compared with the control;** b** enriched pathways among the down-regulated DEGs associated with long-term acidic stress compared with the control;** c** enriched pathways among the up-regulated DEGs associated with shock treatment compared with the control;** d** enriched pathways among the down-regulated DEGs associated with shock treatment compared with the control

The identified DEGs were further subjected to pathway functional enrichment analysis, and the top 20 pathways with significant enrichment are shown in Fig. 2. The most enriched pathways associated with the up-regulated DEGs in long-term acidic stress compared with the control included the two-component system, nitrogen metabolism, RNA degradation, porphyrin and chlorophyll metabolism, and ATP-binding cassette (ABC) transporters (Fig. 2a). Moreover, the most enriched pathways of the down-regulated DEGs in long-term acidic stress were associated with glutathione metabolism (Fig. 2b). Regarding the DEGs related to acidic shock treatment, the most enriched pathways among the up-regulated DEGs included biosynthesis of cofactors and ribosome (Fig. 2c), and those among the down-regulated DEGs included oxidative phosphorylation, photosynthesis-antenna proteins, photosynthesis, ABC transporters, nitrogen metabolism, and amino and nucleotide sugar metabolism (Fig. 2d). Further investigation is required to fully understand the impact of these pathways on the underlying mechanisms of acid responses in *S. elongatus* PCC 7942.

### Validation of acidic stress-responsive genes in *S. elongatus* PCC 7942

To further validate acidic stress-responsive genes in *S. elongatus* PCC 7942, 42 genes out of the DEGs identified above were selected due to their significant fold changes in the above transcriptomics and KEGG pathway enrichment analyses, and single gene knockout analyses were conducted.

**Fig. 3:**
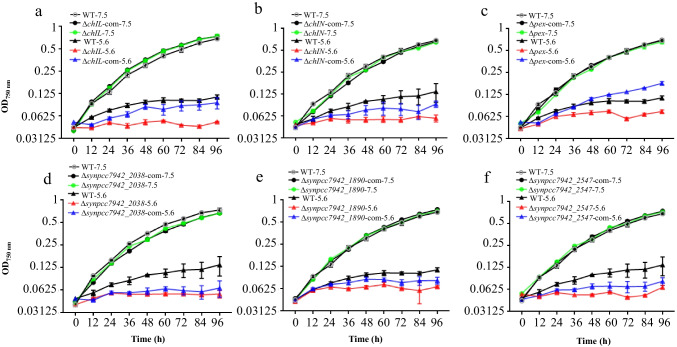
Growth patterns of gene knockout mutants compared with the control and relevant gene complementary analysis in normal BG11 medium and BG11 medium at pH 5.6. **a** *chlL*; **b** *chlN*; **c** *pex*; **d** *synpcc7942_2038*;** e** *synpcc7942_1890*; **f** *synpcc7942_2547*. The error bars represent the calculated standard deviation of the measurements of three biological replicates

The ORFs of 42 targeted genes were individually replaced with a chloramphenicol-resistance cassette in the WT strain to obtain single-gene knockout mutants. Then, the 42 single-gene knockout mutant library was screened for changes in acid stress tolerance compared with that of the WT in normal BG11 medium at pH 7.5 and BG11 medium at pH 5.6. Moreover, to further verify the involvement of specific genes in acid tolerance, the genes that were knocked out were introduced back into the corresponding knockout mutants under the control of the promoter Pcpc560 (Zhou et al. 2014) via natural transformation, and whether the acid tolerance of the resulting strains was restored was further investigated. These efforts led to the identification of six mutants (*chlL* (*synpcc7942_1419*), *chlN* (*synpcc7942_1420*), *pex* (*synpcc7942_0677*), *synpcc7942_2038*, *synpcc7942_1890*, and *synpcc7942_2547* knockouts) (Table 2) involved in acid tolerance. The comparative growth analysis showed that in normal BG11 medium at pH 7.5, all six single-gene knockout mutants and the corresponding complementation strains grew equally as well as the WT, indicating that the knockout and complementation of these genes had no negative effect on cell growth under normal pH conditions (Fig. 3). However, BG11 medium at pH 5.6 significantly inhibited the growth of the six single-gene knockout mutants compared with that of the WT, indicating that the acid tolerance decreased upon the knockout of these six genes individually. These findings indicate that these genes are involved in acid tolerance, which was further confirmed by the partial or complete restoration of acid tolerance to the WT level in the corresponding complementation strains (Fig. 3).

**Table 2:** DEGs identified to be involved in acid tolerance

Function cluster	Gene ID	Fold change		Gene description
		Long-term vs. control	Shock vs. control	
Porphyrin and chlorophyll metabolism	*chlL*	3.53	0.37	Ferredoxin: protochlorophyllide reductase (ATP-dependent) iron-sulfur ATP-binding protein
	*chlN*	2.52	0.18	Ferredoxin: protochlorophyllide reductase (ATP-dependent) subunit N
Transcription regulator	*pex*	2.01	2.09	Helix-turn-helix transcriptional regulator
	*synpcc7942_2038*	2.56	2.58	Helix-turn-helix transcriptional regulator
Hypothetical protein	*synpcc7942_1890*	4.17	2.048	Unknown
	*synpcc7942_2547*	3.16	2.7	Unknown

### Engineering of acid tolerance by overexpressing acidic stress-responsive genes in *S. elongatus* PCC 7942

**Fig. 4:**
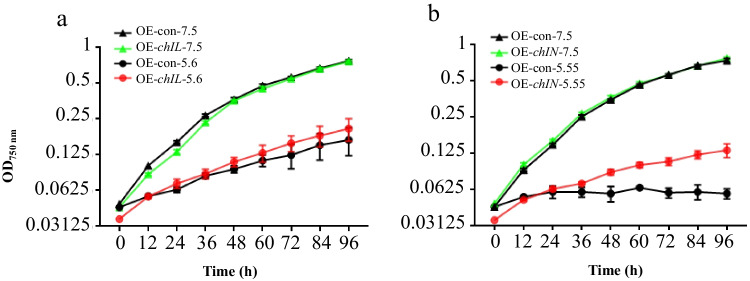
Growth patterns of control and relevant overexpression mutants in BG11 medium at pH 5.6 or 5.55. **a** *chlL*; **b** *chlN*. The error bars represent the calculated standard deviation of the measurements of three biological replicates

To further engineer the acid tolerance of *S. elongatus* PCC 7942, the six acidic stress-responsive genes identified above were individually overexpressed in the WT strain. The acid tolerance analysis showed that overexpression of either *chIL* or *chIN* successfully improved the acid tolerance of WT at pH 5.6 or 5.55, respectively (Fig. 4a, 4b). The enhanced acid tolerance could confer *S. elongatus* PCC 7942 potential enhanced primary productivity and more robustness, rendering it an excellent chassis for synthetic biology.

## Discussion

This study employed RNA-seq to explore DEGs under acidic stress to reveal the resistance mechanism against acidic stress in the model cyanobacterium *S. elongatus* PCC 7942. Overall, 459 up-regulated genes and 468 down-regulated genes were identified under long-term acidic stress conditions compared to those under control conditions; and 1170 DEGs, including 544 up-regulated genes and 626 down-regulated genes, were identified in the comparison of acidic shock treatment with the control. The subsequent KEGG pathway enrichment analysis revealed the top 20 significantly enriched pathways (Fig. 2). Among these significantly enriched pathways were the following DEGs.

### DEGs related to the two-component system

The two-component system was among the up-regulated enriched pathways in long-term acidic stress compared to the control (Fig. 2a), and a total of 12 genes (Table S5) related to the two-component system were up-regulated, including *cikA* (*synpcc7942_0644*) and *pixJ*_*se*_ (*synpcc7942_0858*). CikA is crucial in regulating bacterial circadian rhythms and is responsive to external environmental signals that influence the cellular redox state (Mutsuda et al. 2003; Zhang et al. 2006). PixJ_Se_ senses blue and green light and is important for positive phototactic motility, which is essential for *Synechococcus elongatus* UTEX 3055 to optimize its photosynthetic efficiency (Yang et al. 2018). Here, our results showed that CikA and PixJ_Se_ were involved in acid responses, indicating that these two genes might be involved in multiple functions responding to external environmental signals.

### DEGs related to nitrogen metabolism

Nitrogen metabolism was among the up-regulated enriched pathways in long-term acidic stress compared to the control (Fig. 2a), with seven genes (Table S5) up-regulated. Nitrogen metabolism is crucial for maintaining the carbon/nitrogen (C/N) metabolic balance in cyanobacteria, and it has the potential to address the issue of eutrophication in aquatic systems (Esteves-Ferreira et al. 2018; Yang et al. 2019). Many filamentous cyanobacteria reduce atmospheric dinitrogen to ammonia (Böhme 1998), while nonnitrogen fixers depend on nitrate uptake. Regarding the seven up-regulated genes here, the *NrtABCD* complex was involved in nitrate uptake and transport (Omata 1995), including the nitrate transport ATP-binding proteins NrtD (*synpcc7942_1236*) and NrtC (*synpcc7942_1237*), the nitrate transport permease NrtB (*synpcc7942_1238*), and the nitrate transporter NrtA (*synpcc7942_1239*). The up-regulation of this cluster was reported to be beneficial for intracellular pH stability through ammonia production in acidic stress (Guan and Liu 2020), consistent with our results that these seven genes are involved in the responses to acidic stress in *S. elongatus* PCC 7942. The detailed mechanism remains to be further investigated.

### DEGs related to RNA degradation

RNA plays a central role in protein processing, folding, and assembly and is ubiquitous in all species. In general, RNA transcription and active degradation systems coexist to obtain steady-state RNA levels under stable conditions; however, conditions are not always stable (Houseley and Tollervey 2009), and cells optimize protein expression relative levels to adapt to altered environmental conditions by modulating RNA transcription and degradation (Roy and Chanfreau 2014). In this study, RNA degradation was up-regulated in long-term acidic stress compared with the control, involving a total of six genes (Table S5; Fig. 2a), including RNA helicases *recQ* (*synpcc7942_1301*), *synpcc7942_0685*, *groL* (*synpcc7942_2313*), and *dnaK* (*synpcc7942_2468*). The RNA helicase gene *recQ* was significantly up-regulated (approximately 6.5-fold, Table S2). ATP-dependent RNA helicases are known to be important cofactors associated with RNA processing and degradation (Houseley and Tollervey 2009); furthermore, RNA helicases play a role in abiotic stress (Owttrim 2006) and have been used as tools to modulate plant stress responses (Pandey et al. 2020). Consistent with these results, our results showed that RNA helicases may also play a role in responses to acid stress. Furthermore, the *synpcc7942_0685* gene encodes chaperonin Cpn60/TCP-1 (*groEL* in bacteria), the *groL* gene encodes the GroEL stacked ring complex, and the *dnaK* gene encodes the molecular chaperone DnaK. It has been shown that a lack of GroES/EL renders cells sensitive to osmotic pressure, whereas thermal tolerance is not present in *Caulobacter crescentus* lacking DnaK/J (Susin et al. 2006). Here, our results show that *synpcc7942_0685*, *groE*, and *dnaK* may also be related to responses to acidic stress in *S. elongatus* PCC 7942.

### DEGs related to the ABC transporter pathway

The ABC transporter pathway was among the up-regulated enriched pathways in long-term acidic stress compared to the control, and 15 up-regulated ABC transporter genes (Table S5) were identified (Fig. 2a). ABC transporters are a major membrane-associated transport system that relies on the hydrolysis of ATP to translocate various substrates (Rees et al. 2009). ABC transporters are widely present in gram-negative bacteria, especially cyanobacteria (Tahara et al. 2012). Research has shown that ABC transporters play an essential role in protecting the cell from adverse environmental conditions and are closely related to membrane tolerance to abiotic stresses since they pump out toxic compounds and maintain cellular homeostasis (Dahuja et al. 2021). Overexpression of transporters has been shown to enhance acid stress tolerance in *Lactococcus lactis* NZ9000 (Zhu et al. 2019), suggesting that the up-regulation of ABC transporter proteins potentially contributes to the improved resistance to acidic stress. Among them, *synpcc7942_1126* encodes a permease of the ABC-2 type transporter, a basic component of biofilm formation (Suo et al. 2012), the basic structure by which cells resist acid stress (Guan and Liu 2020).

It has been reported that the responses of *S. elongatus* PCC 7942 to various abiotic stresses encompass multiple mechanisms (Vayenos et al. 2020). For instance, studies on adaptive laboratory evolution have revealed that strains with cadmium tolerance also exhibit resistance to high light intensities of 200 μmol photons m^−2^ s^−1^, although the exact mechanism remains unclear (Xu et al. 2018). In our KEGG pathway analysis, exposure to acidic stress resulted in the up-regulation of numerous genes potentially involved in responding to diverse stress conditions. Previous studies have indicated that many acid-responsive genes are also induced by salt, osmotic, heat, or light stress (Cimdins et al. 2014; Ohta et al. 2005). Additionally, M. A. Sinetova and D. A. Los proposed categorizing stress into two types: heat shock and cold shock, and both of these stress types are associated with reactive oxygen species (ROS) (Sinetova and Los 2016). The generation of ROS is inevitable for aerobic organisms (Latifi et al. 2009), and high salt stress has been shown to increase ROS levels within cells, leading to cellular damage (Yang et al. 2020). In this study, our transcriptomic analysis also suggested that acid stress elicited a complex response in cyanobacteria involving a wide range of cellular processes and molecular pathways.

By single-gene knockout analysis, six genes (*chIL*, *chIN*, *pex*, *synpcc7942_2038*, *synpcc7942_1890*, and *synpcc7942_2547*) were found to be involved in acid tolerance. Among these six acidic stress-responsive genes identified above, the *chIL* encodes the iron-sulfur ATP-binding protein of the light-independent protochlorophyllide reductase, and *chIN* encodes the N subunit of the light-independent protochlorophyllide reductase. Our results showed that (Fig. 3a, 3b) both *chIL* and *chIN* are essential for the acid tolerance of *S. elongatus* PCC 7942. Previous studies have demonstrated that inactivating *chlN* in *Synechocystis* sp. PCC 6803 leads to the absence of chlorophyll and photosystems when the mutant is cultivated under dark conditions (Liu et al. 2005). Additional research has demonstrated the impact of acid rain on photosynthetic activities and plant growth (Debnath et al. 2021). Furthermore, in cyanobacteria, chlorophyll exhibits distinct phenotypes in responses to various environmental stress conditions. For instance, under high-light stress, cyanobacteria adjust chlorophyll content to mitigate potential photodamage resulting from excessive energy absorption and subsequent photoinhibition (Kopecná et al. 2012; Georg et al. 2014). Iron, a crucial element in photosynthesis, significantly influences chlorophyll synthesis, and its availability directly impacts this process (Michel et al. 2003). In environments with iron scarcity, cyanobacteria regulate cellular photosynthetic efficiency by optimizing the amount of photosystem I (PSI) complexes (Georg et al. 2017) and expressing the chlorophyll-binding protein IsiA (Jia et al. 2021). Under UV-B irradiation stress, moderate exposure induces chlorophyll bleaching. However, the bleaching phenotype can recover to pre-irradiation levels after 4–7 days of exposure (He et al. 2002). Under salt stress conditions, the chlorophyll content in cyanobacteria is influenced by various factors, including the environment and the morphology of cyanobacteria (Rezayian et al. 2019). Considering our findings, it can be reasonably speculated that both *chlN* and *chlN* might play a significant role in responses to acid tolerance by mediating the activity of the photosynthetic system, which remains to be further investigated in the future.

The mutants with *pex* or *synpcc7942_2038* knocked out exhibited impaired growth under pH 5.6 conditions (Fig. 3c, 3d). The *pex* encodes a circadian elongator that forms dimers in vivo and acts as a direct negative regulator of *kaiA*. Inactivation of *pex* has been demonstrated to result in the abnormal aggregation of *kaiA* mRNA and a shortened circadian cycle (Kutsuna et al. 2007). Previous studies have also indicated that circadian control systems are involved in the responses to osmotic stress (Nanatani et al. 2014). Given our findings, it is reasonable to deduce that the decrease in acid tolerance in the knockout strain of *pex* might be attributable to the disruption of the normal circadian rhythm cycle in *S. elongatus* PCC 7942. *Synpcc7942_2038*, a member of the xenobiotic response element (XRE) family with a cupin sensor domain, was also shown to be essential for acid resistance (Fig. 3d). Through a comparison using NCBI BLAST, *Synpcc7942_2038* was found to share similarity to the HTH-type transcriptional regulator SutR in *Escherichia coli*. SutR regulates a set of genes involved in different stages of sulfur utilization (Yamamoto et al. 2015). In *Corynebacterium glutamicum*, sulfur assimilation has been demonstrated to directly impact acid tolerance (Xu et al. 2019). Therefore, it is reasonable to deduce that *synpcc7942_2038* might achieve its involvement in acid resistance by regulating sulfur utilization pathways in *S. elongatus* PCC 7942. Two other genes, *synpcc7942_1890* and *synpcc7942_2547*, encoding hypothetical proteins, were also found to be involved in acid tolerance (Fig. 3e, 3f). Further studies are required to determine the specific functions of these proteins in the acid stress response.

Finally, in this study, overexpression of *chIL* or *chIN* individually in WT successfully improved the acid tolerance of WT under acidic stress conditions, while the overexpression of the remaining four genes had no significant effect (Fig. S3), potentially because the expression of these four genes in the WT strain was already saturated; thus, overexpression could not further improve the acid tolerance of the WT strain.

This study provides valuable information that improves our understanding of the acid resistance mechanism in *S. elongatus* PCC 7942 and novel insights for tolerance engineering of cyanobacterial chassis.

## Data availability

All data generated or analyzed during this study are included in this published article and its Additional files. The datasets presented in this study can be found online with accession number PRJNA1026714 (https://www.ncbi.nlm.nih.gov/sra/PRJNA1026714).

## Supplementary information

Below is the link to the electronic supplementary material.

Supplementary file1 (XLSX 233 KB)

Supplementary file2 (PDF 358 KB)

## Declarations

**Ethical approval**

This article does not contain any studies with human participants or animals performed by any of the authors.
