# Supplementary material for: Identification of acidic stress-responsive genes and acid tolerance engineering in Synechococcus elongatus PCC 7942
Source: Appl Microbiol Biotechnol. 2024 Jan 10;108:115. doi: 10.1007/s00253-023-12984-5 (PMC10781874; doi:10.1007/s00253-023-12984-5)
Supplement: Supplementary file 1 [file 253_2023_12984_MOESM2_ESM.pdf]

- **Journal name**

Applied Microbiology and Biotechnology

- **Manuscript Title**

Identification of acidic stress responsive genes and acid tolerance engineering in *Synechococcus elongatus* PCC 7942

- **The name(s) of the author(s)**

Jie Zhang [1, 2, 3], Tao Sun [1, 2, 3, 4], Weiwen Zhang [1, 2, 3, 4], Lei Chen [1, 2, 3*]

- **The affiliation(s) and address(es) of the author(s)**

[1] *Laboratory of Synthetic Microbiology, School of Chemical Engineering & Technology, Tianjin University, Tianjin 300072, P.R. China；*

[2] *Frontiers Science Center for Synthetic Biology, Tianjin University, Tianjin 300072, P.R. China；*

[3] *Key Laboratory of Systems Bioengineering (MOE), Tianjin University, Tianjin 300072, P.R. China；*

[4] *Center for Biosafety Research and Strategy, Tianjin University, Tianjin 300072, P.R. China.*

- **The e-mail address, telephone and fax numbers of the corresponding author**

*Corresponding author:

Prof. Dr. Lei Chen

Laboratory of Synthetic Microbiology; School of Chemical Engineering & Technology; Tianjin University, Tianjin 300072, P.R. China

Tel: 0086-22-2740-6394; Fax: 0086-22-2740-6364; Email: lchen@tju.edu.cn

Fig. S1 Biological reproducibility of RNA-seq data with three distinct clusters formed in the principal component analysis.

Fig. S2 Relative gene expression levels obtained from RNA-seq and qRT-PCR analysis. a) long-term acidic stress VS control; b) shock treatment VS control.

Fig. S3 Growth patterns of overexpression mutants and control in BG11 medium at pH7.5 and pH5.6. a) *pex*; b) *synpcc7942_2038*; c) *synpcc7942_1890*; d) *synpcc7942_2547*. The error bars represented the calculated standard deviation of the measurements of three biological replicates.

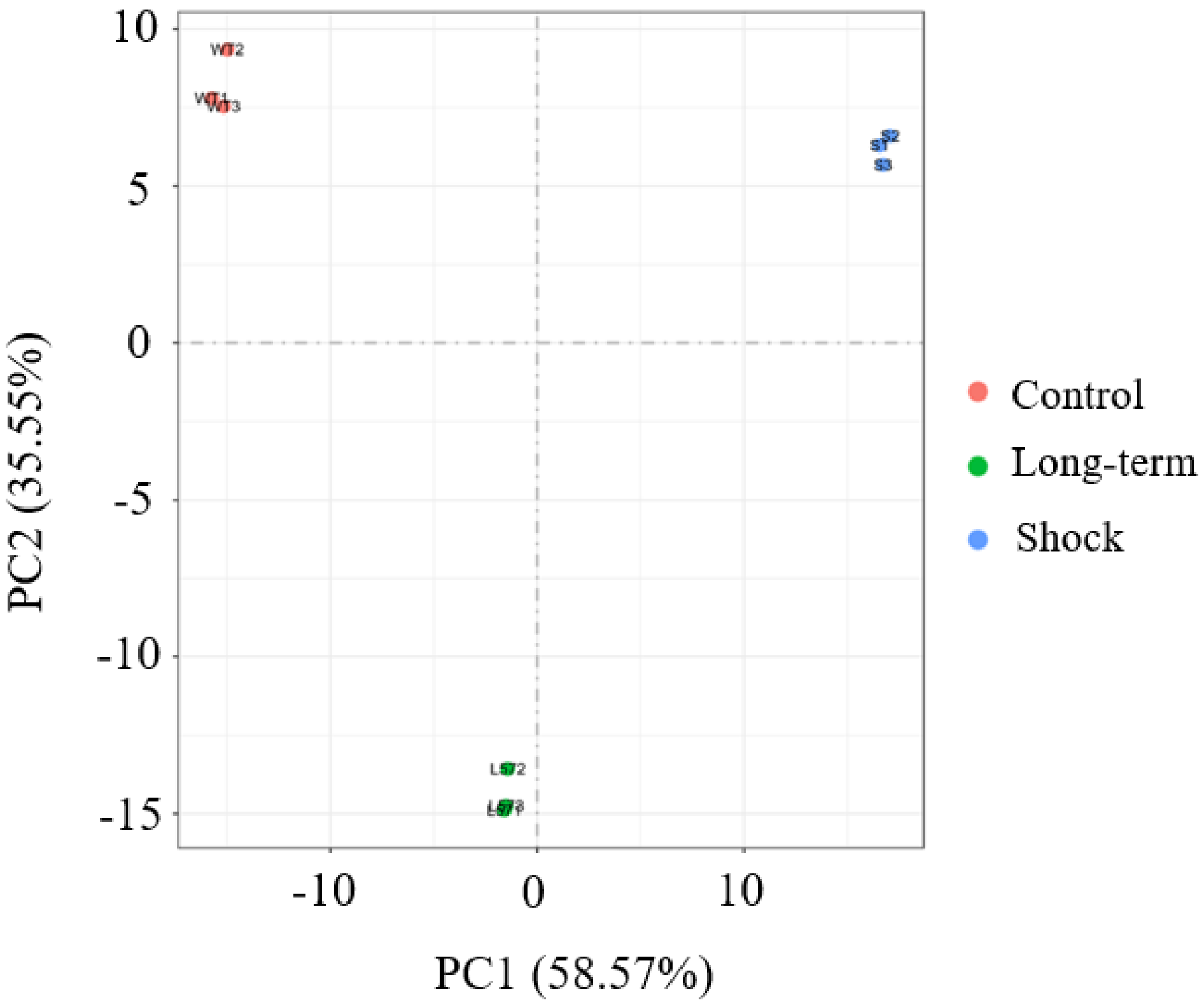

Fig. S1

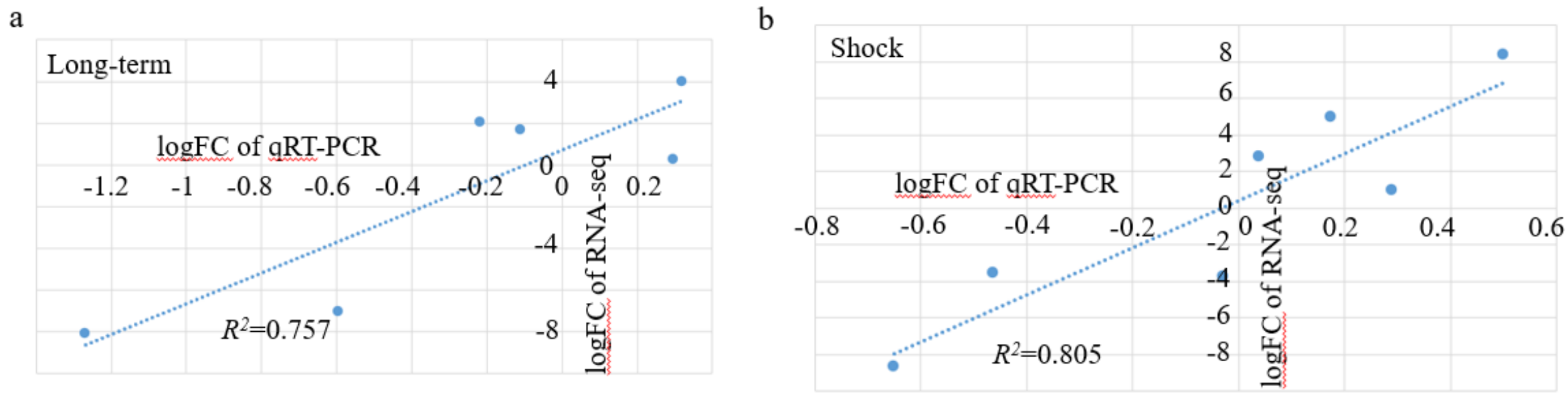
a
Long-term
logFC of qRT-PCR
logFC of RNA-seq
R^2=0.757
-1.2
-1
-0.8
-0.6
-0.4
-0.2
0
0.2
4
0
-4
-8
b
Shock
logFC of qRT-PCR
logFC of RNA-seq
R^2=0.805
-0.8
-0.6
-0.4
-0.2
0
0.2
0.4
0.6
8
6
4
2
0
-2
-4
-6
-8

Fig. S2

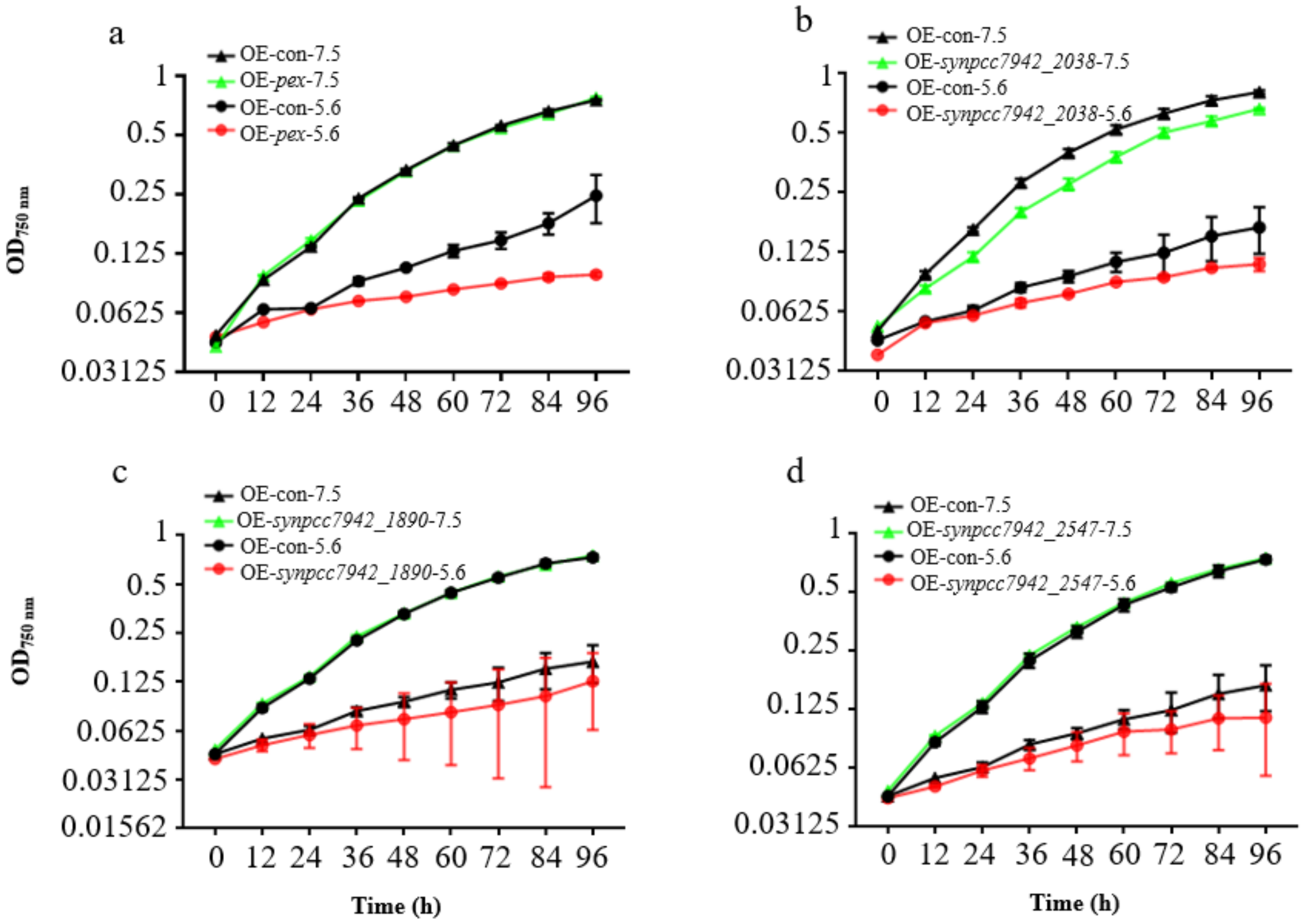
a
OE-con-7.5
OE-pex-7.5
OE-con-5.6
OE-pex-5.6
b
OE-con-7.5
OE-synpcc7942_2038-7.5
OE-con-5.6
OE-synpcc7942_2038-5.6
c
OE-con-7.5
OE-synpcc7942_1890-7.5
OE-con-5.6
OE-synpcc7942_1890-5.6
d
OE-con-7.5
OE-synpcc7942_2547-7.5
OE-con-5.6
OE-synpcc7942_2547-5.6
OD750 nm
Time (h)
1
0.5
0.25
0.125
0.0625
0.03125
0.01562
0 12 24 36 48 60 72 84 96

Fig. S3

Credit Author Statement

J. Z wrote the manuscript, conducted the experiments and analyzed the data. L. C edited the manuscript and T. S supervised the work. Funding was sourced by L. C and W.W. Z.
